# The Effect of Different Statin‐Based Lipid‐Lowering Strategies on C‐Reactive Protein Levels in Patients With Stable Coronary Artery Disease

**DOI:** 10.1002/clc.24301

**Published:** 2024-06-19

**Authors:** Zhimin Xue, Miao Ye, Hangpan Jiang, Duanbin Li, Xulin Hong, Zhezhe Chen, Ya Li, Binquan Zhou, Wenbin Zhang, Miaoyun Wang

**Affiliations:** ^1^ Department of Cardiology, Sir Run Run Shaw Hospital, School of Medicine Zhejiang University Zhejiang Hangzhou China; ^2^ Key Laboratory of Cardiovascular Intervention and Regenerative Medicine of Zhejiang Province Zhejiang Hangzhou China; ^3^ Department of Cardiology, The Fourth Affiliated Hospital, College of Medicine Zhejiang University Zhejiang China; ^4^ Hangzhou Medical College Affiliated Lin An People's Hospital Hangzhou China

**Keywords:** ezetimibe, inflammation, lipid‐lowering therapy, stable coronary artery disease, statin

## Abstract

**Background:**

Statins are lipid‐lowering drugs with favorable anti‐inflammatory effects. This study aimed to explore different statin‐based lipid‐lowering strategies to reduce high‐sensitivity C‐reactive protein (hs‐CRP).

**Hypothesis:**

The hypothesis is that different statin‐based lipid‐lowering strategies might reduce hs‐CRP.

**Methods:**

This retrospective study included 3653 patients who underwent percutaneous coronary intervention (PCI). Three statin‐based lipid‐lowering strategies were investigated, including different types of statins (atorvastatin vs. rosuvastatin), statin combined with ezetimibe therapy (vs. without), and intensive statin therapy (vs. regular). The hs‐CRP levels and blood lipid indicators were measured at baseline and after 1‐month lipid‐lowering therapy. Multivariable linear regression analysis and structural equation mode analysis were conducted to verify the association between different lipid‐lowering strategies, Δhs‐CRP (%) and ΔLDL‐C (%).

**Results:**

Totally, 3653 patients were enrolled with an average age of 63.81 years. Multivariable linear regression demonstrated that statin combined with ezetimibe therapy was significantly associated with decreased Δhs‐CRP (%) (*β* = −0.253, 95% CI: [−0.501 to −0.005], *p* = 0.045). The increased ΔLDL‐C (%) was an independent predictor of elevated levels of Δhs‐CRP (%) (*β* = 0.487, 95% CI: [0.15−0.824], *p* = 0.005). Furthermore, structural equation model analysis proved that statin combined with ezetimibe therapy (*β* = −0.300, *p* < 0.001) and intensive statin therapy (*β* = −0.032, *p* = 0.043) had an indirect negative effect on Δhs‐CRP via ΔLDL‐C.

**Conclusions:**

Compared with routine statin use, statin combined with ezetimibe therapy and intensive statin therapy could further reduce hs‐CRP levels.

## Graphical abstract

This study included stable coronary artery disease patients who underwent PCI and following treatment with statins. During treatment, hs‐CRP, LDL‐C, high‐density lipoprotein cholesterol, non‐HDL‐C, total cholesterol, and triglycerides were measured at baseline and 1 month of lipid‐lowering therapy. In conclusion, statin combined with ezetimibe therapy and intensive statin therapy could further reduce hs‐CRP levels by lowering LDL‐C levels.

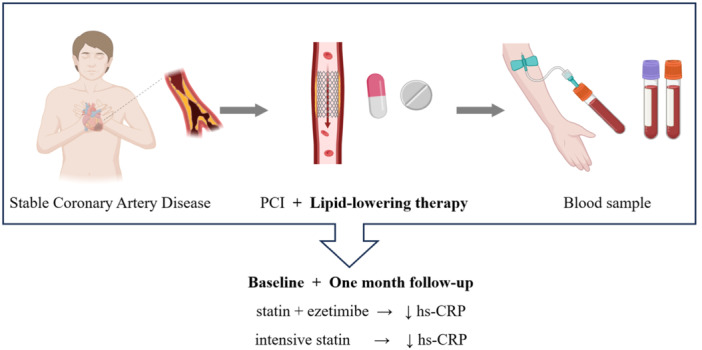

**Abbreviations**
CAD: coronary artery disease
CK: creatine kinase
Cr: creatinine
CRP: C‐reactive protein
eNOS: endothelial nitric oxide synthase
HDL‐C: high‐density lipoprotein cholesterol
HIS: hospital information system
hs‐CRP: high‐sensitivity CRP
LDL‐C: low‐density lipoprotein‐cholesterol
NO: nitric oxide
PCI: percutaneous coronary intervention
SCAD: stable coronary artery disease
TC: total cholesterol
TG: triglycerides

## 1 Introduction

Coronary artery disease (CAD) is the most prevalent cause of mortality worldwide, and stable coronary artery disease (SCAD) is one of the most common clinical manifestations [1]. For decades, lipid‐lowering therapy has been a mainstay for CAD patients [2]. American College of Cardiology/American Heart Association (ACC/AHA) 2019 guidelines recommended lipid‐lowering therapy as the primary and secondary prevention of CAD [3]. Statins are the commonly used lipid‐lowering drugs, which primarily reduce the low‐density lipoprotein cholesterol (LDL‐C), delay arteriosclerosis, and decrease the risk of cardiovascular adverse events [4]. In addition to the lipid‐lowering effect, statins have pleiotropic vasculoprotective properties, such as reducing inflammation and improving endothelial function [5]. The anti‐inflammatory effect of statins is currently a hot research topic.

Inflammation plays a crucial role in the pathogenesis of atherosclerosis, so anti‐inflammatory therapy is vital for a good prognosis in CAD patients [6, 7]. C‐reactive protein (CRP) is a widely used inflammatory biomarker, and elevated CRP is related to the higher incidence of cardiovascular diseases [8]. High‐sensitivity CRP (hs‐CRP) has been reported to be a better predictor of inflammatory response and vascular disorders than CRP [9]. Numerous studies have demonstrated that statins have an anti‐inflammatory property that significantly reduces hs‐CRP levels [10, 11]. Previous research solely focused on validating that different stain‐based lipid‐lowering therapies involving different types of statins, such as statin combined with ezetimibe, could decrease the levels of CRP or hs‐CRP [12, 13]. However, it has not been adequately studied whether such an effect on hs‐CRP differs significantly between different statin‐based lipid‐lowering strategies. It is also unclear whether statin‐based lipid‐lowering strategies directly reduce hs‐CRP levels or are indirectly mediated by LDL‐C.

Therefore, the purpose of this study is to compare the effect of different statin‐based lipid‐lowering strategies, including different types of statins (atorvastatin vs. rosuvastatin), statin combined with ezetimibe therapy (vs. without), and intensive statin therapy (vs. regular), on the decrease of hs‐CRP in patients treated with statins after percutaneous coronary intervention (PCI) and to further explore whether this effect on hs‐CRP decrease is mediated by LDL‐C.

## 2 Materials and Methods

### 2.1 Study Design

This research was an observational retrospective study that followed the Strengthening the Reporting of Observational Studies in Epidemiology (STROBE) [14]. From May 2013 to October 2018, CAD patients who underwent PCI following treatment with statins in Sir Run Run Shaw Hospital were studied. The inclusion criteria were as follows: (1) patients of SCAD who underwent PCI and were treated with oral lipid‐lowering medications (atorvastatin or rosuvastatin with/without ezetimibe) after discharge, (2) hs‐CRP and LDL‐C were evaluated on admission and 1‐month follow‐up after discharge, and (3) data of demographic, laboratory examination, and medication was available for analysis. A total of 4937 individuals were available for analysis according to the above inclusion criteria. The exclusion criteria were as follows: (1) patients who were treated with other statins such as simvastatin and pravastatin (*N* = 977), (2) patients with hs‐CRP > 10 mg/L at baseline or follow‐up (*N* = 69), (3) patients with clinically apparent infectious diseases during hospitalization or 1‐month follow‐up (*N* = 37), (4) patients with familial hyperlipidemia (*N* = 178), and (5) patients who died during 1‐month follow‐up (*N* = 23). Finally, a total of 3653 patients were enrolled.

### 2.2 Definitions

According to the 2019 ACC/AHA guideline [3], regular statin therapy was defined as the administration of atorvastatin 20 mg per day or rosuvastatin 10 mg per day, and intensive statin therapy was defined as the administration of atorvastatin (40 mg or greater) or rosuvastatin (20 mg or greater). Statin combined with ezetimibe therapy was defined as the regular dose of statin with ezetimibe of 10 mg or greater per day.

This study elected Δhs‐CRP (%) and ΔLDL‐C (%) to represent the change of hs‐CRP levels. Δhs‐CRP (%) and ΔLDL‐C (%) were the ratio of change in hs‐CRP and LDL‐C from baseline to 1‐month follow‐up, calculated as the (follow‐up minus baseline) divided by baseline, respectively.

In this study, the median level of Δhs‐CRP (%) was −18.6%. Patients were classified into two groups depending on the median level: high‐effective Δhs‐CRP (%) group (Δhs‐CRP (%) < −18.6%) and low‐effective Δhs‐CRP (%) group (Δhs‐CRP (%) ≥ −18.6%). The plus sign (+) indicated an increase in the hs‐CRP levels after 1 month of lipid‐lowering therapy, whereas the minus sign (–) indicated a decrease in the hs‐CRP levels.

### 2.3 Data Collection

The data were collected from the hospital information system. The demographic information included age, gender, comorbidity, medication at discharge, and follow‐up records. During treatment, hs‐CRP, LDL‐C, high‐density lipoprotein cholesterol (HDL‐C), non‐HDL‐C, total cholesterol (TC), and triglycerides (TG) were measured at baseline and 1 month of lipid‐lowering therapy. Besides, the levels of creatine kinase (CK) and creatinine (Cr) were also recorded at baseline and 1‐month follow‐up. All blood samples were obtained after fasting overnight for at least 12 h and measured by experienced operators in a central laboratory.

### 2.4 Statistical Analysis

Continuous variables were shown as the mean ± standard deviation and were compared by Mann−Whitney *U* tests. Categorical variables were represented as counts (proportions) and were compared by the *χ* ^2^ test or Fisher's exact test (if the expected cell value was <5). Spearman's correlation analysis was determined and visualized by a correlation matrix plot. A loess smooth curve was depicted to visualize the relationship between ΔLDL‐C (%) and Δhs‐CRP (%). The univariable and multivariable linear regression analyses were performed to assess the influence of different lipid‐lowering strategies on both ΔLDL‐C (%) and Δhs‐CRP (%), respectively. Furthermore, the structural equation model was conducted to determine the direct and indirect effects through ΔLDL‐C (%) of lipid‐lowering strategies on the Δhs‐CRP (%) levels.

A two‐tailed *p* < 0.05 was considered significant. Statistical analysis was performed using SPSS software, version 18.0 (SPSS Inc., Chicago, IL, USA), and R, version 3.5.1 (The R Foundation for Statistical Computing, Vienna, Austria).

## 3 Results

### 3.1 Population Characteristics on the Baseline and 1‐Month Follow‐up

A total of 3653 patients were enrolled. The average age was 63.81 years, and the male population accounted for 69.4%. According to the median change in Δhs‐CRP (%) levels, patients were divided into the following two groups: high‐effective Δhs‐CRP (%) group (*n* = 1828) and low‐effective Δhs‐CRP (%) group (*n* = 1825).

Baseline demographic characteristics and laboratory data are summarized in Table 1. As compared to the low‐effective Δhs‐CRP (%) group, patients in the high‐effective Δhs‐CRP (%) group had a higher level of baseline hs‐CRP (2.68 ± 2.28 vs. 1.25 ± 1.28 mg/L, *p* < 0.001) and baseline LDL‐C (2.30 ± 0.95 vs. 2.22 ± 0.94 mmol/L, *p* < 0.005). As for lipid‐lowering strategies, patients in the high‐effective Δhs‐CRP (%) group tended to receive more statin combined with ezetimibe therapy (55.6% vs. 44.3%, *p* < 0.001), while there was no significant difference in different types of statins (51.1% vs. 48.9%, *p* = 0.288) or intensive stain therapy (51.4% vs. 48.6%, *p* = 0.539) between groups.

**Table 1:** Baseline characteristics.

Characteristics	Overall (*n* = 3653)	High‐effective Δhs‐CRP (%) group (*n* = 1828)	Low‐effective Δhs‐CRP (%) group (*n* = 1825)	*p* value
Demographic features				
Age, years old	63.81 ± 10.44	63.60 ± 10.73	64.07 ± 10.15	0.375
Male, *n* (%)	2535 (69.4)	1272 (69.6)	1263 (69.2)	0.830
Diabetes, *n* (%)	774 (21.2)	403 (22.0)	371 (20.3)	0.219
Hypertension, *n* (%)	2059 (56.4)	1016 (55.6)	1043 (57.2)	0.356
Smoking (yes/quit/no), (%)	64.1/15.3/20.7	63.2/15.1/21.7	64.9/15.5/19.7	0.312
Drinking (yes/quit/no), (%)	73.0/9.2/17.7	72.8/9.1/18.2	73.4/9.3/17.4	0.818
Laboratory data				
Baseline hs‐CRP (mg/L)	1.95 ± 1.98	2.68 ± 2.28	1.25 ± 1.28	<0.001*
Baseline LDL‐C (mmol/L)	2.26 ± 0.94	2.30 ± 0.95	2.22 ± 0.94	0.005*
Baseline HDL‐C (mmol/L)	1.07 ± 0.29	1.09 ± 0.29	1.06 ± 0.29	0.357
Baseline non‐HDL‐C (mmol/L)	3.13 ± 0.82	3.11 ± 0.79	3.14 ± 0.83	0.657
Baseline TC (mmol/L)	4.19 ± 1.20	4.20 ± 1.21	4.17 ± 1.20	0.380
Baseline TG (mmol/L)	1.72 ± 1.13	1.71 ± 1.15	1.72 ± 1.11	0.699
Baseline CK (U/L)	145.75 ± 277.38	167.46 ± 334.49	124.68 ± 205.46	0.168
Baseline Cr (μmol/L)	78.21 ± 35.95	78.30 ± 42.62	78.18 ± 27.93	0.839
Lipid‐lowering strategies				
Rosuvastatin (vs. atrovastatin)	1552 (42.5)	793 (51.1)	759 (48.9)	0.288
Statin with ezetimibe (vs. without)	735 (20.1)	409 (55.6)	326 (44.3)	0.001*
Intensive statin (vs. regular)	486 (13.3)	250 (51.4)	236 (48.6)	0.539

*Note:* Data are presented as *n* (%) or mean ± SD.

Abbreviations: CK, creatine kinase; Cr, creatinine; HDL‐C, high‐density lipoprotein‐cholesterol; hs‐CRP, high‐sensitivity C‐reactive protein; LDL‐C, low‐density lipoprotein‐cholesterol; TC, total cholesterol; TG, triglycerides.

* *p* < 0.05.

After 1‐month of treatment, the follow‐up outcomes are displayed in Table 2. The high‐effective Δhs‐CRP (%) group had lower blood lipid levels, including LDL‐C (1.60 ± 0.60 vs. 1.66 ± 0.61 mmol/L, *p* < 0.001), non‐HDL‐C (2.29 ± 0.62 vs. 2.40 ± 0.65 mmol/L, *p* < 0.001), and TC (3.42 ± 0.79 vs. 3.52 ± 0.84 mmol/L, *p* < 0.001). Besides the levels of HDL (1.12 ± 0.32 vs. 1.09 ± 0.31 mmol/L, *p* = 0.432), CK (100.12 ± 55.76 vs. 100.62 ± 88.01 U/L, *p* = 0.198), and Cr (81.50 ± 35.20 vs. 80.85 ± 26.23 μmol/L, *p* = 0.871), there were no significant difference in the two groups. However, patients in the high‐effective Δhs‐CRP (%) group had lower follow‐up hs‐CRP levels (1.00 ± 0.95 vs. 2.15 ± 2.00 mg/L, *p* < 0.001).

**Table 2:** The outcome of 1‐month follow‐up.

Characteristics	Overall (*n* = 3653)	High‐effective Δhs‐CRP (%) group (*n* = 1828)	Low‐effective Δhs‐CRP (%) group (*n* = 1825)	*p* value
Follow‐up hs‐CRP, mg/L	1.57 ± 1.66	1.00 ± 0.95	2.15 ± 2.00	<0.001*
Δhs‐CRP, %	41 ± 290	−57 ± 22	139 ± 385	<0.001*
Follow‐up LDL‐C, mmol/L	1.63 ± 0.61	1.60 ± 0.60	1.66 ± 0.61	<0.001*
ΔLDL‐C percent, %	−21 ± 29	−24 ± 29	−18 ± 30	<0.001*
Follow‐up HDL‐C mmol/L	1.10 ± 0.30	1.12 ± 0.32	1.09 ± 0.31	0.432
Follow‐up non‐HDL‐C, mmol/L	2.34 ± 0.62	2.29 ± 0.62	2.40 ± 0.65	<0.001*
Follow‐up TC, mmol/L	3.47 ± 0.82	3.42 ± 0.79	3.52 ± 0.84	<0.001*
Follow‐up TG, mmol/L	1.47 ± 0.85	1.45 ± 0.80	1.49 ± 0.90	0.187
Follow‐up CK, U/L	100.38 ± 73.94	100.12 ± 55.76	100.62 ± 88.01	0.198
Follow‐up Cr, μmol/L	81.09 ± 31.00	81.50 ± 35.20	80.85 ± 26.23	0.871

*Note:* Data are presented as *n* (%) or mean ± SD.

Abbreviations: CK, creatine kinase; Cr, creatinine; HDL‐C, high‐density lipoprotein‐cholesterol; hs‐CRP, high‐sensitivity C‐reactive protein; LDL‐C, low‐density lipoprotein‐cholesterol; TC, total cholesterol; TG, triglycerides.

* *p* < 0.05.

### 3.2 The Correlation Analysis of Statin‐Based Lipid‐Lowering Strategies, ΔLDL‐C (%) and Δhs‐CRP (%)

Spearman's correlation analysis was performed to determine the association between lipid‐lowering strategies, ΔLDL‐C (%) and Δhs‐CRP (%) (Supporting Information S1: Figure 1). The correlation matrix showed that the statin combined with ezetimibe therapy was positively correlated with LDL‐C levels at baseline (*r* = 0.34) and negatively correlated with ΔLDL‐C (%) (*r* = −0.30). Furthermore, the relationship between ΔLDL‐C (%) and Δhs‐CRP (%) was determined and visualized by the loess smooth curves in Supporting Information S1: Figure 2. The result demonstrated an underlying linear association between ΔLDL‐C (%) and Δh.

### 3.3 The Influence of Statin‐Based Lipid‐Lowering Strategies on Δhs‐CRP (%)

To evaluate the efficacy of Δhs‐CRP (%) of different lipid‐lowering strategies, a linear regression analysis was performed to estimate the linear relationship among different lipid‐lowering strategies and Δhs‐CRP (%) in patients treated with statins after PCI (Table 3). The univariant linear regression analysis demonstrated that age, smoke, ΔLDL‐C (%), and statin combined with ezetimibe therapy were associated with the levels of Δhs‐CRP (%) (*p* < 0.1). Multivariable linear regression verified that statin combined with ezetimibe therapy was significantly associated with the Δhs‐CRP (%) (*β* = −0.253, 95% CI [−0.501 to −0.005], *p* = 0.045), while this association was absent when incorporating ΔLDL‐C (%) into the model (*β* = −0.151, 95% CI [−0.409 to 0.106], *p* = 0.250), which indicated that this effect was partly because of LDL‐C levels. The results also showed that the increased ΔLDL (%) was an independent predictor of elevated Δhs‐CRP (%) (*β* = 0.487, 95% CI: [0.15−0.824], *p* = 0.005).

**Table 3:** Linear regression results of lipid‐lowering strategies and △LDL‐C (%) on △hs‐CRP (%).

	Univariable linear regression		Multivariable linear regression		Multivariable linear regression^a^	
	B [95% CI]	*p* value	B [95% CI]	*p* value	B [95% CI]	*p* value
Age	0.013 [0.004 to 0.022]	0.006*	0.01 [0.001 to 0.02]	0.036*	0.011 [0.002 to 0.02]	0.022*
Male	−0.041 [−0.239 to 0.158]	0.689				
Diabetes	−0.185 [−0.415 to 0.045]	0.115				
Hypertension	0.053 [−0.137 to 0.242]	0.586				
Smoke	−0.109 [−0.225 to 0.007]	0.065	−0.069 [−0.186 to 0.048]	0.250	−0.065 [−0.182 to 0.052]	0.278
Drink	−0.075 [−0.196 to 0.046]	0.226				
△LDL‐C (%) rosuvastatin	0.576 [0.257 to 0.894]	<0.001*	0.487 [0.15 to 0.824]	0.005*		
(vs. atorvastatin) statin with ezetimibe	−0.169 [−0.359 to 0.022]	0.082	−0.112 [−0.306 to 0.081]	0.255	−0.118 [−0.312 to 0.075]	0.230
(vs. without) intensive statin	−0.238 [−0.473 to −0.004]	0.046*	−0.151 [−0.409 to 0.106]	0.250	−0.253 [−0.501 to −0.005]	0.045*
(vs. regular)	0.198 [−0.079 to 0.475]	0.160	0.135 [−0.14 to 0.41]	0.335	0.122 [−0.153 to 0.397]	0.385

*Note:* Variables to be entered into the multivariable linear regression analysis were chosen on the basis of the results of univariable analysis (*p* < 0.1).

Abbreviations: hs‐CRP, high‐sensitivity C‐reactive protein; LDL‐C, low‐density lipoprotein‐cholesterol.

^a^ Multivariable linear regression excluded the ΔLDL‐C (%) for further analysis.

* *p* < 0.05.

However, the connection between Δhs‐CRP (%) and different types of statins strategy (*β* = 0.135, 95% CI: [−0.14 to 0.41], *p* = 0.335) or intensive statin therapy strategy (*β* = −0.112, 95% CI: [−0.306 to 0.081], *p* = 0.255) was not significant.

### 3.4 The Influence of Statin‐Based Lipid‐Lowering Strategies on ΔLDL‐C (%)

As shown in Table 4, the linear regression analysis was performed to determine the influence of lipid‐lowering strategies on ΔLDL‐C (%). The univariable linear analysis demonstrated that a total of five variables were significantly associated with ΔLDL‐C (%), including age, sex, diabetes, hypertension, and statin combined with ezetimibe therapy (all *p* < 0.05). Then, multivariable linear regression analysis showed that the levels of ΔLDL‐C (%) were negatively correlated with statin combined with ezetimibe therapy (*β* = −0.204, 95% CI: [−0.230 to −0.178], *p* < 0.001) and intensive statin therapy (*β* = −0.028, 95% CI: [−0.056 to −0.001], *p* = 0.046). However, the decrease of ΔLDL‐C (%) was independent of different types of statins (*β* = −0.011, 95% CI: [−0.032 to 0.009], *p* = 0.272).

**Table 4:** Linear regression results of lipid‐lowering strategies on ΔLDL‐C (%).

	Univariant linear regression		Multivariant linear regression	
	*B* [95% CI]	*p* value	*B* [95% CI]	*p* value
Age	0.002 [0.001 to 0.003]	<0.001*	0.002 [0.001 to 0.003]	<0.001*
Male	0.030 [0.008 to 0.052]	0.008*	0.035 [0.013 to 0.057]	0.002*
Diabetes	0.047 [0.024 to 0.070]	<0.001*	0.033 [0.009 to 0.057]	0.006*
Hypertension	0.027 [0.008 to 0.046]	0.006*	0.019 [−0.002 to 0.041]	0.075
Smoke	0.003 [−0.009 to 0.015]	0.630		
Drink	−0.001 [−0.013 to 0.011]	0.853		
Rosuvastatin (vs. atrovastatin)	−0.014 [−0.033 to 0.005]	0.158	−0.011 [−0.032 to 0.009]	0.272
Statin with ezetimibe (vs. without)	−0.218 [−0.240 to −0.195]	<0.001*	−0.204 [−0.230 to −0.178]	<0.001*
Intensive statin (vs. regular)	−0.012 [−0.040 to 0.016]	0.392	−0.028 [−0.056 to −0.001]	0.046*

*Note:* Variables to be entered into the multivariable linear regression analysis were chosen on the basis of the results of the univariable analysis.

Abbreviation: LDL‐C, low‐density lipoprotein cholesterol.

* *p* < 0.05.

### 3.5 Structural Equation Modeling Analysis

Based on the association between lipid‐lowering strategies, Δhs‐CRP (%) and ΔLDL‐C (%), we hypothesized that the lipid‐lowering strategies might exert the effect on Δhs‐CRP (%) through two pathways: the direct pathway (statin‐based lipid‐lowering strategies → Δhs‐CRP [%]) and the indirect pathway (statin‐based lipid‐lowering strategies → ΔLDL‐C [%] → Δhs‐CRP [%]). Thus, the structural equation model analysis was performed to verify this hypothesis (Supporting Information S1: Figure 3 and Table 5). The results showed that all three lipid‐lowering strategies had no direct effect on Δhs‐CRP (%) (all *p* > 0.05). However, ΔLDL‐C (%) was positively related to the levels of Δhs‐CRP (%) (*β* = 0.054, *p* = 0.002). Statin combined with ezetimibe therapy (*β* = −0.300, *p* < 0.001) and intensive statin therapy (*β* = −0.032, *p* = 0.043) had significantly negative correlations with ΔLDL‐C (%). The link between statin combined with ezetimibe therapy and ΔLDL‐C was stronger than that of intensive statin therapy (nearly 10 times). This structural equation model analysis revealed a mediation effect of ΔLDL‐C (%) on the relationship between lipid‐lowering strategies (statin combined with ezetimibe therapy and intensive statin therapy) and Δhs‐CRP (%).

**Table 5:** Results of structural equation modeling analysis.

Hypothesis			Standardized path coefficient	Standard error	*p* value	Support
Rosuvastatin (vs. atrovastatin)	→	ΔLDL‐C (%)	−0.019	0.009	0.239	No
Statin with ezetimibe (vs. without)	→	ΔLDL‐C (%)	−0.300	0.012	<0.001*	Yes
Intensive statin (vs. regular)	→	ΔLDL‐C (%)	−0.032	0.014	0.043*	Yes
Rosuvastatin (vs. atrovastatin)	→	Δhs‐CRP (%)	−0.026	0.097	0.109	No
Statin with ezetimibe (vs. without)	→	Δhs‐CRP (%)	−0.015	0.125	0.386	No
Intensive statin (vs. regular)	→	Δhs‐CRP (%)	0.023	0.141	0.173	No
ΔLDL‐C (%)	→	Δhs‐CRP (%)	0.054	0.170	0.002*	Yes
Statin with ezetimibe (vs. without)	↔	Rosuvastatin (vs. atrovastatin)	0.023	0.003	0.163	No
Rosuvastatin (vs. atrovastatin)	↔	Intensive statin (vs. regular)	−0.025	0.003	0.127	No
Statin with ezetimibe (vs. without)	↔	Intensive statin (vs. regular)	−0.056	0.002	<0.001*	Yes

Abbreviations: hs‐CRP, high‐sensitivity C‐reactive protein; LDL‐C, low‐density lipoprotein cholesterol.

* *p* < 0.05.

## 4 Discussion

The current study demonstrated that different statin‐based lipid‐lowering strategies exhibited different effects on decreasing hs‐CRP levels. The LDL‐C partly mediated the relationship between statin‐based lipid‐lowering strategies and hs‐CRP levels. We found that hs‐CRP reduction and LDL‐C reduction occur simultaneously and in parallel correlation when statins are combined with ezetimibe therapy, and our hypotheses and conclusions partially demonstrate a possible mediating effect. However, it is important to note that our study was not about LDL‐C and hs‐CRP but about the potential effect of lipid‐lowering strategies on the presence of hs‐CRP, and therefore, this paper cannot prove a causal relationship between LDL‐C and hs‐CRP. Compared to routine statin use, statin combined with ezetimibe therapy and intensive statin therapy could further reduce hs‐CRP levels. There was no significant difference in the effects on hs‐CRP among different types of statins. Lipid‐lowering therapy, particularly statin therapy, has been the cornerstone of CAD prevention due to its potent lipid‐lowering effect [15]. Recently, researchers found that statin therapy was also a promising anti‐inflammatory therapy [16]. The JUPITER trial, a landmark trial, proved that statin therapy contributed to a significant reduction in hs‐CRP level, decreasing the risk of cardiovascular events [12]. Tan et al. reported that the combination therapy with atorvastatin and ezetimibe efficiently controlled the variation of LDL‐C and, in the meantime, alleviated the inflammation, becoming an alternative approach in clinical practice [17]. This study further compared the efficacy of different stain‐based lipid‐lowering strategies on hs‐CRP levels based on previous research. This study demonstrated that compared to statin monotherapy, statin combined with ezetimibe therapy was significantly associated with reducing hs‐CRP. Therefore, statin combined with ezetimibe therapy in clinical practice might be a more encouraging option for downregulating inflammation in patients after PCI. However, no differences were observed among different types of statins or between intensive statin therapy and regular statin therapy.

More importantly, LDL‐C partly mediated the relationship between statin‐based lipid‐lowering strategies and hs‐CRP levels, as hs‐CRP reduction and LDL‐C reduction occur simultaneously, and in parallel correlation, when statins are combined with ezetimibe therapy as shown in Supporting Information S1: Figure 3, the association of therapy, LDL‐C, and hs‐CRP was significant. LDL‐C is the important determinant of arteriosclerosis in various lipid markers with a proinflammatory effect [18, 19]. Miller et al. found that oxidized LDL‐C can result in severe inflammatory processes [20]. Consistently, this study established a close relationship between hs‐CRP and LDL‐C. The multivariable linear regression analysis showed that statin combined with ezetimibe therapy was significantly associated with the Δhs‐CRP (%), while this association was absent when incorporating ΔLDL‐C (%) into the model, suggesting that the decrease of LDL‐C strongly influenced such effects on hs‐CRP reduction. Structural equation model analysis also proved that ΔLDL‐C (%) played an intermediary role. In this study, although intensive statin therapy also had the indirect pathway (intensive statin therapy → ΔLDL‐L [%] → Δhs‐CRP [%]), the correlation between intensive statin therapy and LDL‐C was weak, which might explain why intensive statin therapy was not significantly linked with the decrease of hs‐CRP levels when compared with regular statin therapy.

Based on the available studies, the effect of statin‐based lipid‐lowering strategies on lowering hs‐CRP levels may be clarified by the following mechanisms. First, the inflammation usually cooccurs with oxidative stress, an important component of atherosclerosis pathogenesis [21, 22]. Hyperlipidemia exacerbates this oxidative stress, thereby aggravating the inflammatory status and promoting the progression of atherosclerotic plaques [23]. Consequently, applying lipid‐lowering strategies can lower blood lipid levels and reduce the hs‐CRP levels by improving oxidative stress. Second, PCI procedures may cause mechanical injury to the coronary vessels, leading to a sharp increase of hs‐CRP in the short term [24]. To narrow down the effect of PCI surgery on hsCRP, we included all SCAD patients after PCI and used △hsCRP (%) from baseline as an endpoint to convert the absolute value of the change into a relative value, taking into account the individual variability that exists at baseline. Lipid‐lowering drugs, especially statins, can improve vascular endothelial function by activating the endothelial nitric oxide synthase (eNOS) system and increasing the production of nitric oxide (NO) [25]. NO is regarded as an important vascular protective molecule and an important mediator of inflammatory reactions, which can promote the health of blood vessels [26]. Therefore, lipid‐lowering treatment may induce NO production and endothelial repair by employing the anti‐inflammatory effect. Third, the inflammation of atherosclerosis is a chronic systemic inflammatory response rather than a local vascular inflammation [27]. The LDL‐C circulating in the bloodstream can enhance the activation of toll‐like receptors, which subsequently mediate immunological response and increase hs‐CRP levels [28]. It can be speculated that due to the application of lipid‐lowering drugs, the decrease in LDL‐C contributes to preventing the activation of the inflammatory cytokine and alleviating inflammatory reactions [29].

Although important findings were presented here, some limitations still deserve attention. First, as a retrospective study, the inherent selection and follow‐up bias were unavoidable. Second, hs‐CRP may not be a reliable diagnostic marker as various factors can affect the hs‐CRP levels, especially infection or injury. Therefore, this study excluded patients with clinically apparent infectious diseases or abnormally elevated hs‐CRP levels (hs‐CRP > 10 mg/L). Third, we only analyzed the effect of statin and ezetimibe, and other lipid‐lowering drugs had not been investigated. Fourth, the effect of lipid‐lowering strategies on hs‐CRP was studied only at 1 month, and it is unclear whether long‐term efficacy remains unchanged.

## 5 Conclusions

Compared with routine statin use, statin combined with ezetimibe therapy and intensive statin therapy could further reduce hs‐CRP levels. There was no significant difference in the effects on hs‐CRP between different types of statins.

## Author Contributions

Wenbin Zhang, Miaoyun Wang, and Binquan Zhou designed the research study. Zhimin Xue organized these data and drafted the manuscript with the help of Miao Ye, Hangpan Jiang, Duanbin Li, Xulin Hong, and Ya Li. Hangpan Jiang, Zhezhe Chen, and Ya Li analyzed the data. Zhimin Xue drew the pictures. Wenbin Zhang detected any errors in the whole process. All authors contributed to editorial changes in the manuscript. All authors read and approved the final manuscript.

## Ethics Statement

The authors are accountable for all aspects of the work in ensuring that questions related to the accuracy or integrity of any part of the work are appropriately investigated and resolved. The study was conducted in accordance with the Declaration of Helsinki (as revised in 2013). The study was approved by the Ethics Committee of Sir Run Run Shaw Hospital of Zhejiang University (No. 20200803‐34).

## Consent

Individual consent for this retrospective analysis was waived.

## Conflicts of Interest

The authors declare no conflicts of interest.

## Supporting information

Supporting information.

## Data Availability

The data that support the findings of this study are available from the corresponding author upon reasonable request.
