# Supplementary material for: The Effect of Different Statin‐Based Lipid‐Lowering Strategies on C‐Reactive Protein Levels in Patients With Stable Coronary Artery Disease
Source: Clin Cardiol. 2024 Jun 19;47(6):e24301. doi: 10.1002/clc.24301 (PMC11187842; doi:10.1002/clc.24301)
Supplement: Supplementary file 1 — Supporting information. [file CLC-47-e24301-s001.docx]

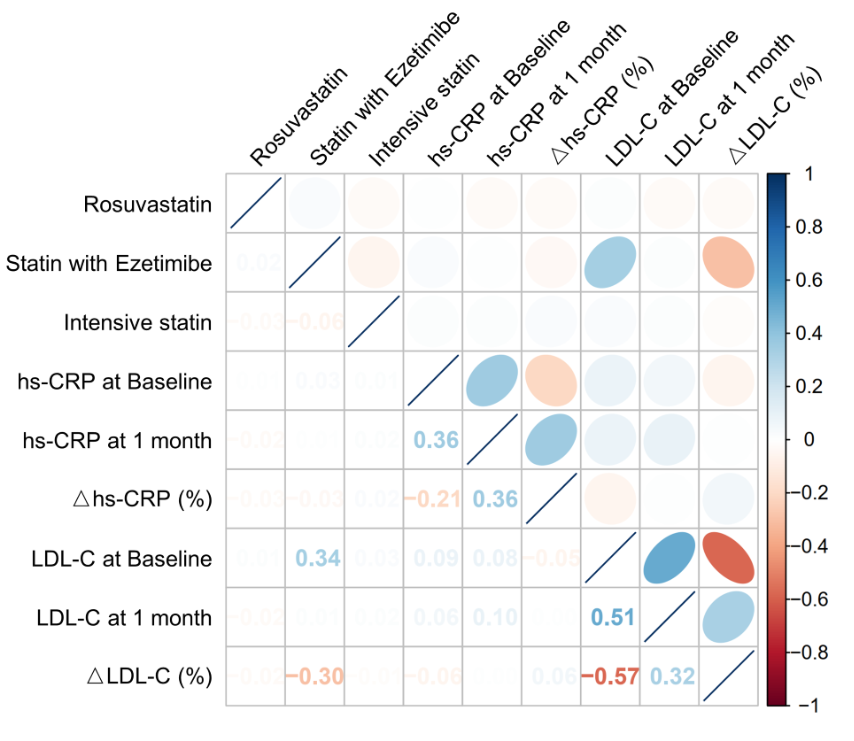


**Fig. S1. Correlation matrix.** Correlation matrix of statin-based lipid-lowering strategies, Δhs-CRP (%), and △LDL-C (%). Pearson correlations are displayed on the bottom-left part of the matrix plot. A higher correlation is represented by lower transparency and narrower ellipses. Blue indicates positive correlation and red indicates negative correlation.


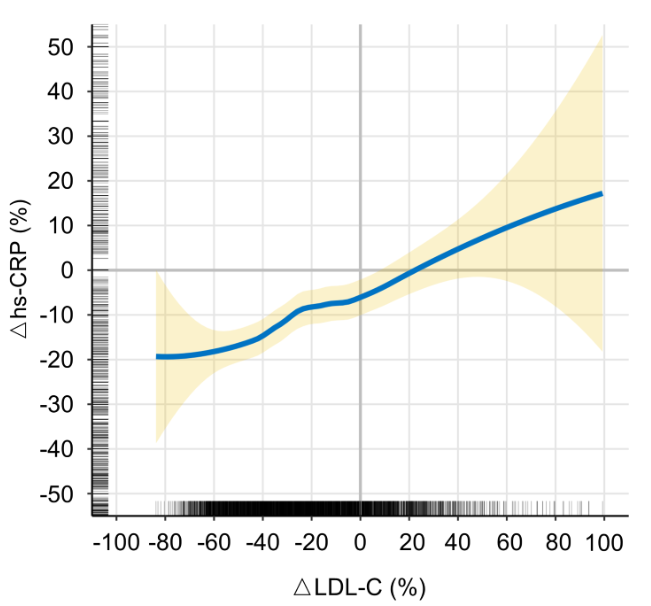


**Fig. S2. Loess smooth curves of △LDL-C (%) with Δhs-CRP (%).** The curves of loess regression (span = 0.8) were used to depict the relationship between ΔLDL-C (%) and Δhs-CRP (%). The semi-transparent ribbon around the solid line indicates 95% confidence interval. Rug plots show the distribution of the variables.


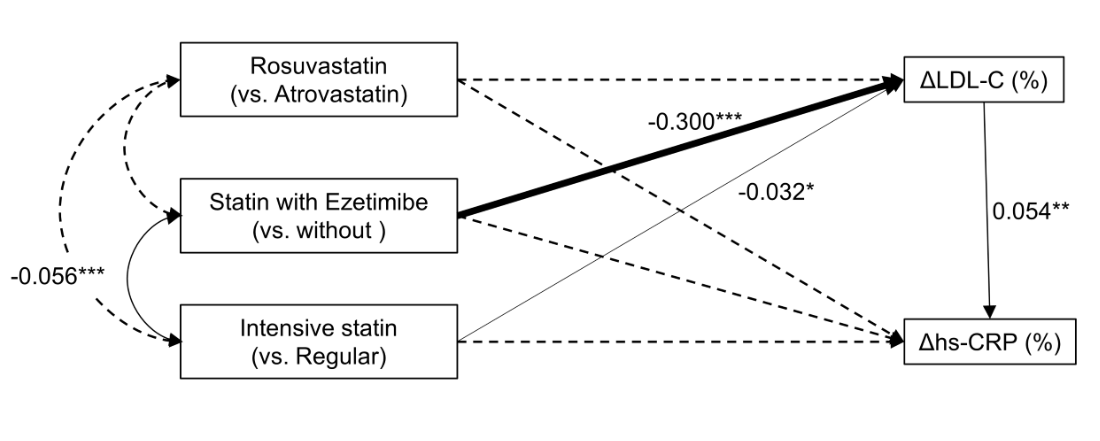


**Fig. S3. Structural equation model diagram.** The structural equation model diagram depicted the relationship between variables by the solid arrows (valid paths) and dashed arrows (invalid paths). The numbers next to the solid arrows show the corresponding regression coefficients between the respective variables in the boxes. The asterisks indicate the levels of significance for the respective regression coefficients: (*) *p* < 0.05; (**) *P* < 0.01; (***) *p* < 0.001. The direction and thickness of the arrows indicate the direction (causality) of the mutual impact of the variables and the approximate values of the regression coefficients, respectively.
